# A Next Generation Semiconductor Based Sequencing Approach for the Identification of Meat Species in DNA Mixtures

**DOI:** 10.1371/journal.pone.0121701

**Published:** 2015-04-29

**Authors:** Francesca Bertolini, Marco Ciro Ghionda, Enrico D’Alessandro, Claudia Geraci, Vincenzo Chiofalo, Luca Fontanesi

**Affiliations:** 1 Department of Agricultural and Food Sciences, Division of Animal Sciences, University of Bologna, Viale Fanin 46, 40127, Bologna, Italy; 2 Department of Veterinary Sciences, Animal Production Unit, University of Messina, Polo Universitario dell'Annunziata, 98168, Messina, Italy; 3 Meat Research Consortium, Polo Universitario dell’Annunziata, 98168, Messina, Italy; Agricultural University of Athens, GREECE

## Abstract

The identification of the species of origin of meat and meat products is an important issue to prevent and detect frauds that might have economic, ethical and health implications. In this paper we evaluated the potential of the next generation semiconductor based sequencing technology (Ion Torrent Personal Genome Machine) for the identification of DNA from meat species (pig, horse, cattle, sheep, rabbit, chicken, turkey, pheasant, duck, goose and pigeon) as well as from human and rat in DNA mixtures through the sequencing of PCR products obtained from different couples of universal primers that amplify 12S and 16S rRNA mitochondrial DNA genes. Six libraries were produced including PCR products obtained separately from 13 species or from DNA mixtures containing DNA from all species or only avian or only mammalian species at equimolar concentration or at 1:10 or 1:50 ratios for pig and horse DNA. Sequencing obtained a total of 33,294,511 called nucleotides of which 29,109,688 with Q20 (87.43%) in a total of 215,944 reads. Different alignment algorithms were used to assign the species based on sequence data. Error rate calculated after confirmation of the obtained sequences by Sanger sequencing ranged from 0.0003 to 0.02 for the different species. Correlation about the number of reads per species between different libraries was high for mammalian species (0.97) and lower for avian species (0.70). PCR competition limited the efficiency of amplification and sequencing for avian species for some primer pairs. Detection of low level of pig and horse DNA was possible with reads obtained from different primer pairs. The sequencing of the products obtained from different universal PCR primers could be a useful strategy to overcome potential problems of amplification. Based on these results, the Ion Torrent technology can be applied for the identification of meat species in DNA mixtures.

## Introduction

The possibility to identify the species of origin of meat and meat products is an important issue to prevent and detect frauds that derive from the economic incentives to substitute premium meat or high added-value products with products of lower quality obtained from cheaper species. Substitutions could be also derived by accidental events and labelling errors. However, all these substitutions can have not only economic implications but may also rise concerns related to food safety and security, lifestyle, religious and ethical aspects and are main objectives in forensics investigations [1].

DNA is particularly suitable for the identification of the species of origin of meat or many other specimens because it contains species-specific information, is stable and can be analysed from processed and cooked products. During the last two decades, quite a large number of DNA based methods have been developed to this purpose. Most of them rely on PCR amplification of informative DNA fragments that are then analysed using different approaches (PCR-RFLP, PCR-RAPD, PCR-AFLP, species-specific PCR, PCR-SSCP, PCR-DGGE, PCR-FINS, high-resolution melting, etc.) [2–6] that usually can detect one species at the time (or just a few). Preferred amplified regions are from mitochondrial DNA (mtDNA) genes such as 12S, 16S, D-loop, cytochrome b or cytochrome c oxidase I (COI) containing conserved regions across species that allow the design of universal primers, and internal sequences that contain species-specific differences. However, these approaches need to know *at priori* what could be the species that might be present in order to apply specific discriminatory analytical steps for their identification, with poor multiplexing detection potential. To overcome these limits, dot-blot and microarray detection systems that can analyse at the same time more than one species have been recently proposed [7, 8]. Despite the increased informativity of these systems, the inherent limit determined by their construction (presence or absence of only some species-specific probes) cannot give the possibility to detect unexpected or unknown species.

Next generation sequencing (NGS) technologies have revolutionized the way to analyse DNA increasing tremendously the throughput and combining DNA sequencing and quantification in a single step [9]. NGS is becoming a standard approach in a large number of studies in all species and in many different fields, including resequencing or *de novo* sequencing of large and small genomes, metagenomics and transcriptomics among many other research and applied areas in which sequence data are needed [10–13]. Sequence analysis in NGS experiments is not based on any limiting supports, as in the case of electrophoretic methods (i.e. gel based systems, like Sanger sequencing) or probe hybridization (i.e. microarray). Flexibility of NGS is obtained through sequence data analysis with appropriate bioinformatics tools [14, 15]. Commercially available benchtop NGS platforms, that could be potentially useful to capture sequence data for species identification, are Illumina, 454 pyrosequencing and Ion Torrent technologies [10, 16, 17]. Ion Torrent platform is based on a semiconductor sequencing technology that can detect small modifications of pH in a chip that occur during the elongation steps in the sequencing process [17]. Advantages of the Ion Torrent platform are due to the low cost per run, the speed of the sequencing step, the possibility to barcode different samples that can run on the same chip and the possibility to use different chips that can allow different scales of sequencing throughput according to the analytical needs [18, 19].

Despite the power of NGS for species identification just few studies have been conducted towards this purpose [20–25] and, to our knowledge thus far no investigation has been reported for meat species determination using the Ion Torrent semiconductor platform.

In this paper, we evaluated the potential of next generation semiconductor based sequencing technology for the identification of meat species (mammals and birds) by sequencing PCR products obtained from different universal primer pairs that amplify mtDNA genes. The study was designed 1) to verify the amplification obtained from three universal primer pairs, 2) to analyse the discriminatory power of the amplified target sequences and the experimental error rates by evaluating NGS reads and then 3) to evaluate the potential and limits of the combination of different mtDNA target sequences to identify some of the most common meat species in DNA mixtures.

## Methods

### Ethics statement

Meat species samples were from common commercialized species. Meat samples were purchased from a local retailers in Bologna. The seven animal species that produced the meat samples were not killed for research. Rat muscle sample was obtained from a naturally deceased animal found in the countryside, in a private owned land in the Province of Bologna. Written permission was provided by the landowner for the collection of this material. The rat was not sacrificed for the purpose of this study. All animals were not treated or killed for the purpose of this study. Human blood was from one author (LF) and written consent was obtained for its use after having consulted the University of Bologna research review board. According to the Italian and European legislation, no ethical committee approval or any other authorization was needed for this study.

### Species and DNA isolation

Eleven meat species (mammals and birds) were included in this study: pig (*Sus scrofa domesticus*), horse (*Equus caballus*), cattle (*Bos taurus*), sheep (*Ovis aries*), rabbit (*Oryctolagus cuniculus*), chicken (*Gallus gallus domesticus*), turkey (*Meleagris gallopavo*), pheasant (*Phasianus colchicus*), duck (*Anas platyrhynchos domesticus*), goose (*Anser anser*), and pigeon (*Columba livia*). In addition, human (*Homo sapiens*) and rat (*Rattus rattus*) were included to evaluate the potential of the designed approach to detect unexpected/potentially contaminating species. DNA was extracted from skeletal muscle tissues from meat samples purchased from retailers (all meat species) and rat or blood (human) using the Wizard Genomic DNA Purification kit (Promega Corporation, Madison, WI, USA), following the manufacturer instructions. DNA quantitation and quality assessment was obtained in triplicate using a Nanophotometer P-330 instrument (Implen GmbH, München, Germany). DNA quality was also evaluated by visual inspection on 1% agarose gel electrophoresis in TBE1X buffer after staining with ethidium bromide. All extracted DNA samples had A_260/280_ >1.8.

### PCR analyses

For the subsequent PCR analyses, DNA samples of the 13 different species were used separately or in pool. DNA pools were prepared as follows (Table 1): a) one pool with equimolar DNA from the seven mammalian species (100 ng for each species) included in the study (pig, horse, cattle, sheep, rabbit, human and rat); b) one pool with equimolar DNA from the six avian species (100 ng for each species) included in the study (chicken, turkey, pheasant, duck, goose and pigeon); c) one pool with equimolar DNA from all 13 species included in the study and its replicate (100 ng for each species; pool c1 and pool c2); d) one pool including DNA from all mammalian species, 5 of which with equimolar DNA (100 ng for each species), whereas for the pig and the horse DNA was 1/10 (10 ng); e) one pool including DNA from all 7 mammalian species, 5 of which with equimolar DNA (100 ng for each species), whereas for the pig and the horse DNA was 1/50 (2 ng). All PCR analyses were carried out using 20 ng of each DNA sample separately for each DNA pool. That means that considering the proportion of DNA included in the different DNA pools, for pool 1 about 2.9 ng of DNA for each species was included in the reaction, for pool 2 about 3.3 ng and for pool 3 about 1.5 ng. For pool 4 and pool 5 the pig and horse DNA included in the reactions were about 0.28 ng and 0.09 ng respectively. Extracted DNA was used at equimolar concentration to mimic for all meat species the real content of the target mtDNA in skeletal muscle, estimating that the mtDNA content from this tissue could be similar for each species, as the purpose of this study was to test the potential of the designed approach on species identification from meat samples. According to this approximation, the number of reads obtained from each species from DNA mixtures containing different amount of DNA (see below) could provide a preliminary quantitative indication [24], even if this experiment was not designed specifically to obtain a precise quantitative evaluation.

**Table 1:** Species composition of the different DNA pools used in PCR analyses.

Pool Code^1^	DNA Pool	Species^2^
a	Mammalian DNA	Pig, horse, cattle, sheep, rabbit, human and rat
b	Avian DNA	Chicken, turkey, pheasant, duck, goose and pigeon
c1	Mammalian + Avian DNA	Pig, horse, cattle, sheep, rabbit, human, rat, chicken, turkey, pheasant, duck, goose and pigeon
c2	Mammalian + Avian DNA	Pig, horse, cattle, sheep, rabbit, human, rat, chicken, turkey, pheasant, duck, goose and pigeon
d	Mammalian DNA 1:10	Pig^3^, horse^3^, cattle, sheep, rabbit, human and rat
e	Mammalian DNA 1:50	Pig^4^, horse^4^, cattle, sheep, rabbit, human and rat

^1^ Pools c1 and c2 were two different replicates of the same pool

^2^ DNA of each species was equimolar (100 ng)

^3^ Pig and horse DNA was 1/10 of that of the other species

^4^ Pig and horse DNA was 1/50 of that of the other species

Primers used for the amplification reactions were from [26] and [27] (Table 2). These primers were designed on the 12S and 16S mitochondrial rRNA genes (thereafter indicated as 12S and 16S respectively) and selected aligning sequences from 122 [26] or 30 [27] mammalian species and successfully tested also in birds, reptiles and fishes [27]. Primer pairs were indicated as 12S_KH and 16S_KH [26] and 16S_Ki [27], respectively (Table 2). PCR amplifications were performed in a 2720 thermal cycler (Life Technologies, Carlsbad, CA, USA) in a total volume of 20 μL that included 20 ng of genomic DNA (as indicated above), 1X of PCR buffer, 10 pmol of each primer, 2.0 mM of MgCl_2_, 2.0 mM of each dNTP, and 1 U of *Taq* DNA Polymerase recombinant (Thermo Scientific-Fermentas, Vilnius, Lithuania) using the following cycling profile: the first denaturation step of 5 min at 94°C; 35 cycles of 30 s at 94°C, 30 s at the indicated annealing temperature (Table 2), and 30 s at 72°C; the final extension step of 7 min at 72°C. The amplified products were analyzed and quantified after 3% agarose gel electrophoresis in TBE 1X buffer and ethidium bromide staining.

**Table 2:** Primer pairs used for DNA amplification [26, 27].

Name	Primers (5' to 3'): forward and *reverse*	Annealing T (°C)	Amplified fragment (bp)^1^	References
12S_KH	CCCAAACTGGGATTAGATACCC	59	215–222	[26]
	*GTTTGCTGAAGATGGCGGTA*			
16S_KH	GACGAGAAGACCCTATGGAGC	59	112–121	[26]
	*TCCGAGGTCGCCCCAACC*			
16S_Ki	GCCTGTTTACCAAAAACATCAC	62	243–249	[27]
	*CTCCATAGGGTCTTCTCGTCTT*			

^1^ The size of the amplified regions length is different in the considered species. The range is reported.

### Sanger sequencing and target regions

To confirm the correspondence between the expected and the obtained amplified fragments, amplicons produced from PCR of DNA of each single species from all primer pairs were sequenced using the Sanger sequencing method. Briefly, PCR products were treated with ExoSAP-IT (USB Corporation, Cleveland, Ohio, USA) and then were labeled with the Big Dye v3.1 kit (Life Technologies) using the same PCR primers of the amplification reactions. Sequencing products, after purification steps, were loaded on an ABI3500 capillary sequencer (Life Technologies). The produced electropherograms were analysed and visually inspected using CodonCode Aligner (CodonCode Corporation, Dedham, MA, USA) and Bioedit (http://www.mbio.ncsu.edu/BioEdit/). Obtained sequences were analysed using BLASTN (http://blast.st-va.ncbi.nlm.nih.gov/Blast.cgi) against the NCBI nr/nt nucleotide collection (20^th^ Sept. 2014) to confirm their match with the corresponding sequence of the same species already available in database. Accession numbers of the sequences used in this comparison is reported in S1 Table. S1, S2 and S3 Figs reports the alignments of the three amplified regions and S2 Table reports the amplified regions based on the reference sequences. To evaluate the discriminatory potential of the target reference mtDNA genes (S1, S2 and S3 Figs), phylogenetic analyses were obtained using MEGA v. 6.0.5 [28]. Maximum Likelihood trees were built using the default options for DNA sequences considering the three different reference regions separately. These reference sequences were used to evaluate the match, alignment and coverage of reads obtained by NGS using different algorithms (see below). In addition, as described in a subsequent paragraph, error rates were calculated using reference sequences corrected after Sanger sequencing (S3 Table).

### Ion Torrent sequencing and data analysis

Ion Torrent sequencing was obtained from six different DNA libraries (1, 2, 3A, 3B, 4 and 5) that were differentiated with six barcodes (Table 3). The six libraries were constructed using the following amplified products, after treatment with ExoSAP-IT (USB Corporation): Library 1: amplicons of the three primer pairs mixed using equal PCR volume and obtained separately from the amplification of genomic DNA from each different species; Library 2: amplicons of the three primer pairs mixed using equal PCR volume and obtained separately from the mammalian DNA pool and from the avian DNA pool (DNA pools a + b); Library 3A: amplicons of the three primer pairs mixed using equal PCR volume and obtained from the DNA pool containing all species (pool c1)); Library 3B: amplicons of the three primer pairs mixed using equal PCR volume and obtained from the replicate DNA pool containing all species (pool c2); Library 4: amplicons of the three primer pairs mixed at the same amplified PCR volume obtained from the mammalian DNA pool containing 1:10 of pig and horse DNA (pool d); Library 5: amplicons of the three primer pairs mixed at the same amplified PCR volume obtained from the mammalian DNA pool containing 1:50 of pig and horse DNA (pool e).

**Table 3:** Libraries prepared and sequenced with Ion Torrent PGM including amplicons obtained from different DNA mixtures.

Library ID	DNA mixtures used in the PCR	Amplicons (primer pairs)	Amplicons (species/DNA mixtures)
1	DNA from each species (no DNA pool)	All primer pairs	Amplicons for all species (obtained separately)
2	2 DNA pools: Mammalian DNA + Avian DNA separately (pool a+b)	All primer pairs	Amplicons from mammalian DNA pool (pool a) + amplicons obtained from avian DNA pool (pool b)
3A	1 DNA pool (including DNA from all species) (pool c2)	All primer pairs	Amplicons from the DNA pool (pool c1)
3B	1 DNA pool (including DNA from all species)–(pool c2)	All primer pairs	Amplicons from the DNA pool (pool c2)
4	1 DNA pool (including only mammalian species): pig and horse DNA = 1/10 (pool d)	All primer pairs	Amplicons from the DNA pool (pool d)
5	1 DNA pool (including only mammalian species): pig and horse DNA = 1/50 (pool e)	All primer pairs	Amplicons from the DNA pool (pool e)

Libraries were prepared following the instructions for Ion Torrent Personal Genome Machine (PGM; Life Technologies) sequencing of short amplicons. Briefly, for each library, 200 ng of amplified DNA was end-repaired and adapter-ligated with a different barcode (six different barcodes were used) using the Ion Xpress Plus Fragment Library and Ion Xpress Barcode Adapters 1–16 kits (Life Technologies). Then each library was quantified by qPCR using a StepOnePlus Real-Time PCR System (Life Technologies) with the Ion Library Quantitation Kit (Life Technologies). Then, the six barcoded libraries were pooled at the same concentration, clonally amplified by emulsion PCR with the Ion One Touch 400 Template kit (Life Technologies), purified and sequenced with the Ion PGM^TM^ Sequencing 400 kit using a Ion 314 v2 chip (Life Technologies), following the manufacturer protocols.

Sequencing obtained a total of 33,294,511 called nucleotides of which 29,109,688 with Q20 (87.43%) in a total of 215,944 reads. The obtained sequenced reads were first automatically processed by the Torrent Suite (TS) v4.1 on the Ion Torrent Server (Life Technologies). Briefly, reads were first grouped according to the different barcodes, then polyclonal and low quality sequences were filtered and adapters and low quality 3’-ends were trimmed from the high quality grouped reads. After the automatic processes, for each barcode, reads were then trimmed from the primer sequences at 5’ and 3’ end using the *trim* function of HOMER [29]. Trimmed reads were first aligned to the pre-built reference sequence with *bwa* according to the *aln* [30], the SW [31] and *mem* (http://bio-bwa.sourceforge.net/) algorithms using default options. Then, only reads with length ≥ 40 nucleotides, mapping quality ≥ 20 were retained. Bam files of the raw and filtered alignments were obtained using Samtools software [32]. The *idxstats* function of Samtools was finally used to count reads aligned to each region of the reference sequences. To evaluate the quality of the alignments, aligned reads were also visually inspected with Integrative Genomic Viewer (IGV) [33]. Since for all species and amplified fragments the reference sequences were known (as obtained by Sanger sequencing), error rates per base were calculated for each species and target gene combination by counting mismatches obtained in the filtered alignments and considering the number of corrected aligned nucleotides obtained from library 1. All the filtering and processing steps were done using Unix environment and Python scripts. Pearson’s correlation was calculated to compare number of reads obtained from different libraries, group of species (mammalian and avian) and primer pair combinations.

Next generation sequencing data were deposited in the EMBL-EBI European Nucleotide Archive (ENA) with the project accession number PRJEB7911.

## Results

### Sanger sequencing verification of the amplified fragments from different species

Three primer pairs were selected from the literature to amplify mtDNA regions (12S and 16S) containing species-specific information [26, 27]. Phylogenetic trees based on the target regions embedded between the two primers for each pair confirmed the informativeness of the selected mtDNA gene fragments for the species included in this study (S4, S5 and S6 Figs). According to the previous works in which these primer pairs were designed and tested [26, 27], amplification of the target mtDNA regions should be expected from mammals as well as birds (DNA from species of both vertebrate classes has been already successfully amplified with the 16S_Ki pair [27]). To verify what was previously reported, we first tested these primers by amplifying DNA from 11 mammalian and avian meat species, as well as from human and rat. All primer pairs amplified a fragment of the expected size at the same PCR conditions in all species (S2 Table), without any detectable unspecific products, as determined by agarose gel electrophoresis (data not shown). Obtained fragments were also confirmed by Sanger sequencing that showed that all 12S and 16S amplicons matched the expected sequences. Obtained sequences from all species were the same as those reported in the reference sequences except from one different position identified in the sheep and rabbit 12S_KH fragments and in the horse 16S_Ki region (S3 Table). This is due to intraspecific variability that has been already reported in mtDNA of many species that does not prevent the attribution of the target amplified regions to the correct species [20].

### Analysis of Ion Torrent sequencing reads: mapping algorithms

**Fig 1:**
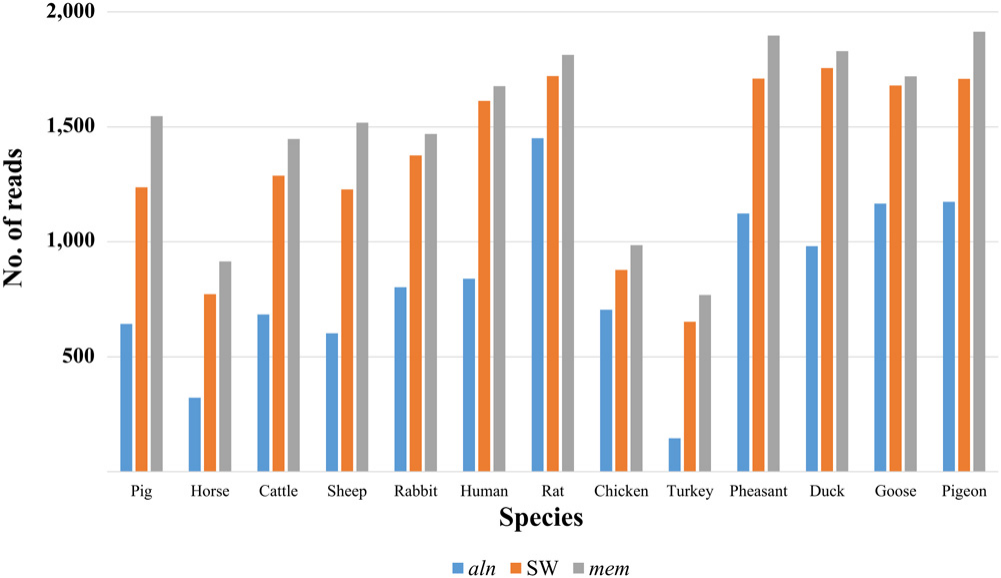
Number of reads mapped by the three algorithms (*aln*, *SW* and *mem*) for each species. The products obtained from the three primer pairs after the filtering step of sequence data obtained from library 1 were considered together.

Amplicons obtained from the separate amplification of DNA of different species were prepared in library 1 and sequenced with the Ion Torrent technology. These data were used to assess the power of filtering and alignment tools without any problems that might derive from un-equal amplification efficiencies among different species and gene regions that could occur during the PCR step on DNA pools including DNA from more than one species (see below). In addition, the possibility to sequence the whole amplicons without any fragmentation made it possible to consider the mapped reads as unbiased estimators of the number of times in which fragments were detected without any additional adjustments that might be needed in case of multi-reads per target region [34]. Three different mapping algorithms included in *bwa* (*aln*, SW and *mem*) were assessed to evaluate their performances in terms of number of mapped reads and quality scores of the alignments (Fig 1). The number of aligned reads to the corresponding reference mtDNA regions (12S_KH, 16S_Ki and 16S_KH), without (raw alignments) and with preliminary filtering steps, is shown in S4 Table. The total number of reads before the filtering step was 27,031; for the raw alignments, *aln* reported 10,902 aligned reads, SW had 20,141 aligned reads and *mem* obtained 20,442 aligned reads. After the filtering steps, 10,637, 17,620 and 19,500 aligned reads were reported for *aln*, SW and *mem*, respectively (S4 Table). Only two combinations (16S_Ki/turkey and 16S_Ki/pigeon) with a number of reads <20 were obtained with the SW and *mem* algorithms. However, it is worth to note that the number of mapped reads obtained for the 16S_Ki fragment was lower for all avian species compared to the mammals, due to a poorer amplification efficiency of these primers in birds (as library 1 was generated from the same amplification volumes for the different primer and “species of origin of DNA template” combinations). If we compare the results obtained by SW and *mem*, the latter algorithm performed better for the purpose of this experiment as for most target region/species combinations it reported a larger number of aligned reads (Fig 1 and S4 Table). Based on these data, we selected the *mem* algorithm for all subsequent analyses.

### Error rates of Ion Torrent reads

Since the reference sequences for all primer pair/species combinations were known (as they were determined by Sanger sequencing) we estimated the error rate per base using all reads obtained in the NGS library that sequenced the pooled amplicons obtained separately from each species (library 1). Reads were first filtered and aligned to the reference sequences using *mem* and error rates were calculated by counting all variants on the aligned sequences (S4 Table). Error rates ranged from 0.0003 (16S_KH/pheasant) to 0.0181 (16S_Ki/human; S4 Table). It was comparable among different sequenced fragment regions (12S_KH = 0.002; 16S_KH = 0.002; 16S_Ki = 0.003) or among species across the three different analyzed mtDNA regions (S5 Table). From these estimations, it seems that error rate is sequence specific as already reported for other NGS technologies (i.e. [20]). These errors might derive from the PCR or from the sequencing steps. In particular, the Ion Torrent sequencing technology cannot resolve correctly homo-polymeric sequences and a large number of the identified errors were in these regions (data not shown). However, as the method is based on the match between reads of the amplified sequences against reference sequences (size of 215–222, 112–121 and 243–249 bp for the three target regions, respectively; Table 2), the average number of less than one error per sequence is irrelevant and does not prevent the correct assignment of the reads to the target region of the correct species.

#### Identification of species-specific reads from the amplification of mixed samples

**Fig 2:**
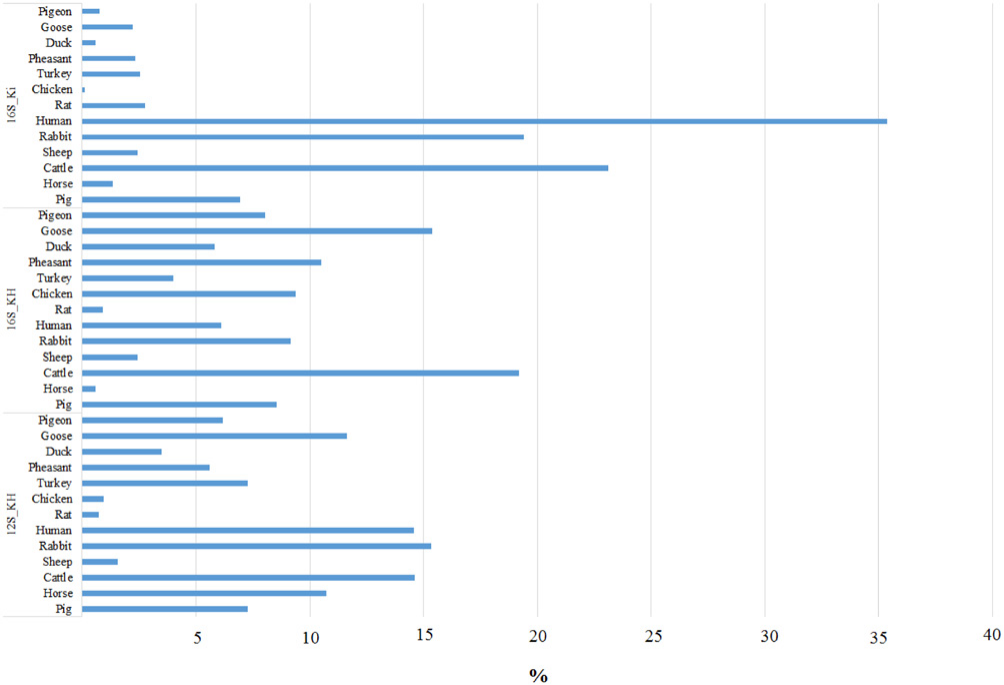
Percentage of reads obtained for the different primer pairs from library 2.

**Fig 3:**
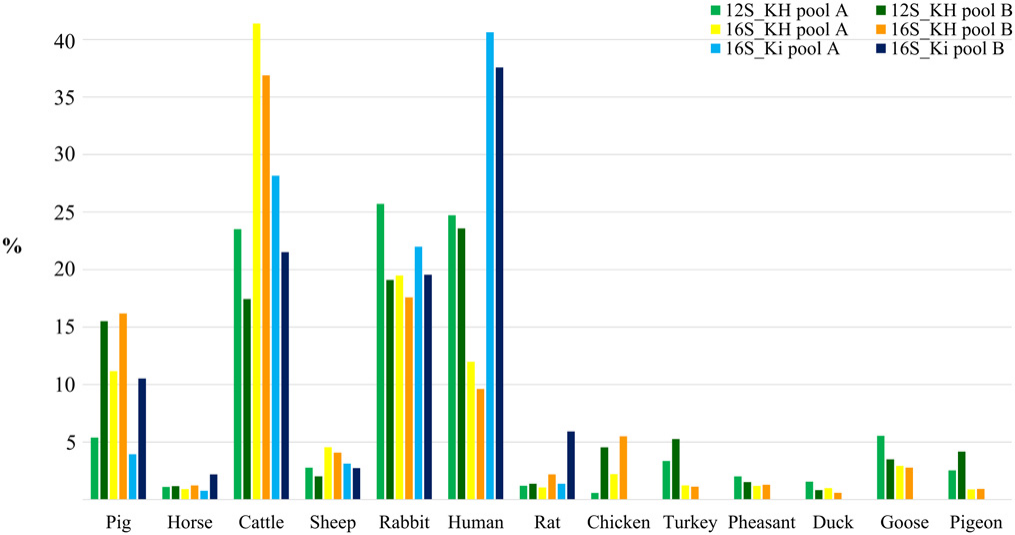
Percentage of reads obtained from libraries 3A and 3B for the different species and primer pairs combinations.

To evaluate the possibility to identify species-specific reads from amplicons derived from DNA mixtures, we first compared the number of reads obtained from library 2 (that contained products separately amplified from the mammalian and avian DNA mixtures, respectively) and libraries 3A and 3B (that contained products amplified from two replicate DNA pools, including both mammalian and avian DNA; S6 Table; Figs 2 and 3). Library 2 produced a total of 24,193 mapped reads and libraries 3A and 3B gave 26,260 and 20,320 mapped reads, respectively. Primer pairs 12S_KH and 16S_KH in libraries 2 and 3A-3B obtained mapped reads for all mammalian and avian species. The proportion of reads mapped to mammalian and avian sequences obtained from the sequencing of amplicons from DNA mixtures containing all species (Libraries 3A and 3B) was similar for both amplified regions (averaged between the two libraries: 12S_KH: mammalian = 0.82 and avian = 0.18; 16S_KH: mammalian = 0.89 and avian = 0.11). Using these two primer pairs, avian fragments were amplified less efficiently compared to mammalian fragments. Similarly, in library 2 primer pair 16S_Ki did not work efficiently on the avian DNA pool compared to the mammalian DNA pool (normalized mean across species based on 1 ng of amplified DNA concentration for each species was 21.6 ± 16.0 and 231.7 ± 232.9 reads for birds and mammals, respectively; S6 Table). This lower efficiency for the avian 16S_Ki region was confirmed by the results of mapped reads obtained from the amplification of the DNA pools containing all species (library 3) in which PCR competition prevented the amplification of avian DNA (among all birds, only one turkey read was mapped in each replicate DNA pool). Among the tested mammalian DNA, the larger number of normalized reads obtained by this primer pair in both library types, 2 and 3A-3B, was obtained for the human and cattle (3577 and 2340 in library 2 and on average 5820.5 and 3762.5 in the two libraries 3, respectively), whereas the lowest number was obtained for the horse in both cases (66.3 and 23.7). Correlation of the number of reads of the same species between the two experiments (library 2 and libraries 3) for the results of primer pair 16S_Ki was 0.99, considering both mammalian data only or mammalian + avian data, providing an indirect evaluation of the experimental repeatability.

The experimental repeatability was evaluated directly comparing the results obtained from libraries 3A and 3B that derives from two replicate DNA pools, obtained from avian and mammalian DNA, amplified separately with the three primer pairs considered in this study (Fig 3). Correlation between the number of reads of the two libraries, considering all primer pair/species combinations, was 0.97 (Table 4). Correlation between the results obtained by different primer pairs indicated that 16S_Ki and 16S_KH produced the highest reproducible results (r > 0.97), whereas the correlation between the 12S_KH products obtained from libraries 3A and 3B was 0.907. This is due to the poorer reproducibility of the avian results that for this primer pair was low (r = 0.204). The overall correlation of the number of reads obtained for the different avian species was in general lower than the correlation obtained for the mammalian species. If we consider that for primer pair 16S_Ki we did not obtain any mapped reads for avian species (only 1 read was obtained for the turkey) and the lower repeatability of the results produced by these primers, the best primer pair was 16S_KH: it produced highly correlated results for mammalian reads and it detected all avian species with the highest correlation between libraries (Table 4). However, from the data obtained from libraries 2 as well as libraries 3A e 3B, it is clear that avian species can be amplified (and then sequenced) less efficiently than mammalian species. This is probably due to the design of the primer pairs that was based on alignments that included only mammalian sequences of 12S and 16S mtDNA [26, 27]. Several mismatches between the primer sequences and the annealed avian target regions (S1, S2 and S3 Figs.) may reduce primer efficiency for these species.

**Table 4:** Pearson’s correlation between the number of mapped reads obtained from libraries 3A and 3B using different primer pairs.

Library 3A	All	All mammals	All avian species	12S_KH	16S_KH	16S_Ki	12S_KH Mammals	12S_KH Avian	16S_KH Mammals	16S_KH Avian	16S_Ki Mammals	16S_Ki Avian
**Library 3B**												
**All**	0.970											
**All mammals**		0.969										
**All avian species**			0.700									
**12S_KH**				0.907								
**16S_KH**					0.983							
**16S_Ki**						0.977						
**12S_KH Mammals**							0.889					
**12S_KH Avian**								0.204				
**16S_KH Mammals**									0.982			
**16S_KH Avian**										0.713		
**16S_Ki Mammals**											0.973	
**16S_Ki Avian**												1.000

All = all species and all primer pairs; All mammals = all primer pairs including only mammalian species; All avian species = all primer pairs including only avian species; when primer pairs are indicated, all species were used in the correlation; the different primer pairs were also considered separately for mammalian and avian species as indicated. Correlation between the results of the two libraries obtained with primer pair 16S_Ki for the avian species is biased due to the lack of sequenced reads in birds.

Using data from libraries 4 and 5, that included amplicons generated from mixtures of mammalian DNA with a reduced quantity of pig and horse DNA, we tested the sensitivity of the different primers to detect DNA from these species whose presence in meat-based products is a matter of concern for some markets/consumers (pig) or for problems derived by recent frauds (horse) [1, 35]. As expected, the number of mapped reads for the 1:10 and 1:50 dilutions obtained with the three primer pairs was lower than what was obtained from libraries 2 and 3A-3B (Table 5 and S6 Table). The 16S_Ki fragments of both species was recovered at both dilutions (no. of reads <20 and <10 for the 1:10 and 1:50 dilutions, respectively). The other two primer pairs were less efficient: for the 1:50 dilution, they did not obtain any reads (16S_KH for the horse) or just few (12S_KH for the horse) and similar situation was for 16S_for the dilution 1:10 in pig (Table 5). If we exclude just these differences for the pig and horse reads, correlation between results obtained from libraries 4 and 5 was 0.99, for all primer pair combinations, further confirming the repeatability obtained from mixtures of mammalian DNA.

**Table 5:** Number of mapped reads for each primer pair and species combinations obtained from libraries 4 and 5 in which pig and horse amplicons were obtained from DNA mixtures in which pig and horse DNA was included at 1:10 and 1:50 ratios, respectively.

Species	12S_KH		16S_KH		16S_Ki	
	Library 4–1:10	Library 5–1:50	Library 4–1:10	Library 5–1:50	Library 4–1:10	Library 5–1:50
Pig	26	7	21	8	30	12
Horse	6	1	1	0	26	17
Cattle	1681	1554	1650	1728	4949	3429
Sheep	193	174	156	196	561	375
Rabbit	1698	1587	746	817	4103	2830
Human	1559	1494	467	558	6430	4596
Rat	98	100	69	84	540	286
Total no. of reads	5261	4917	3110	3391	16639	11545

## Discussion

Other works have evaluated the potential of NGS approaches for species identification. For example, Tillmar et al. [20] tested mammalian universal primers to amplify the 16S gene and sequenced the obtained products with the Roche 454 Junior instrument. Coghan et al. [21] used the same NGS platform to identify, from traditional Chinese medicines, plant and animal DNA after amplification of the *trn*L (plastid) and 16S mtDNA fragments, respectively. In another study on traditional Chinese medicines, plant species were identified with sequence data produced with the 454 GS-Titanium sequencer after amplification of the plant *trn*L and ITS2 conserved regions [23]. A metagenomic approach without any pre-amplification step was simulated and then tested analyzing sausage DNA on Illumina HiSeq 2000 and MiSeq instruments data [24].

In this study we tested the possibility to use the Ion Torrent next generation semiconductor based sequencing technology to identify all species in samples of mixed DNA that included mammalian and avian species. Species identification in meat products is still a hot topic that raises ethical, health and economical concerns generated by frauds and efficient and flexible monitoring analytical systems are needed. The advantage of NGS approaches for species determination derives by the possibility to identify more than one species at the same time, including also un-expected species, without any previous information about their presence, based on obtained reads that can be assigned to different species by matching sequence data already available in databases using bioinformatics tools. In our approach, we first amplified mtDNA regions containing species-specific sequence information by using universal primers reported in the literature and designed on multiple alignments of 12S and 16S mtDNA sequences generated from a large number of mammals [26, 27]. As some of these primers were also successfully tested with avian, reptile and fish DNA [27], we used the three primer pairs to amplify mammalian and avian meat species, including human and rat to evaluate the possibility to identify unexpected or contaminating species that could have forensic values in particular caseworks. Inter-individual sequence variability was not considered in this study as this aspect usually cannot prevent sequence assignments due to the lower level of sequence differences within species than that is present across species. Only in the remote cases in which within species variants would be eventually located in the highly conserved regions in which primers were designed it might be possible to observe different amplification efficiencies among individuals of the same species. On the other hand, inter-individual differences in the amplified regions might be captured by the obtained reads making it possible to deduce information from different animals of the same species that might be present in the analyzed meat samples or specimens. In the sequenced libraries we included the PCR products obtained by three primer pairs demonstrating the possibility to obtain multiplex information from different target regions. This aspect can solve the problem derived by the failure of one primer pair to amplify the DNA from one species or the different amplification efficiency in mixed DNA samples (that we observed between mammalian and avian species with primer pair 16S_Ki) or the presence of individual variants preventing or reducing the amplification of one target region. In addition, different primer pairs could be affected in different ways by the presence of PCR inhibitors when DNA is extracted from complex matrix, including processed food products. The system can be very flexible and additional target regions, including other informative mtDNA genes (i.e. cytochrome b, COI, D-loop), and for each gene more primer pairs that might be useful to differentiate more species or closer species [25], can be amplified and sequenced without any additional costs apart from the limited cost of the PCR. Several other studies have reported the design of universal primers, including also degenerated positions, that could be used to amplify more than one species and comparatively with the same amplification efficiency (i.e. [20, 36–38]). That means that it could be possible to apply this approach not only for the identification of meat species but also for applications in many other fields, including forensic investigations, zoo-archeology, wildlife management and population ecology [39–42]. The short target fragments used in our study (in particular the 16S_KH region) can facilitate the amplification in degraded DNA that can be usually recovered for these investigations. One limit of the technology could be due to the maximum number of reads that can be generated with the three chips available for the Ion Torrent PGM (chip 314, 316 and 318 that can produce up to 10 Mb, 100 Mb and 1 Gb of sequenced DNA, respectively, according to the manufacturer information). This might potentially affect the sensitivity of the assays that is based on the number of reads that can be mapped for each samples and, in turn, the number of samples that can be analyzed in one run using post-PCR barcoding, considering the sensitivity required by each case and samples.

To evaluate the sensitivity of the sequencing assays we analyzed artificial DNA mixtures in which pig and horse DNA was present in a 1:10 or 1:50 ratios (compared to all other mammalian species). The two minor DNA species in both dilutions could be determined with two out of three primer pairs (12S_KH and 16S_Ki). These results confirmed on the one hand the usefulness of using more target regions and on the other hand that this approach is sufficiently sensitive to detect the presence of these two species for field analyses. Based on these results, Ion Torrent can be useful to investigate routinely the presence of these two species in food products. However, additional studies are needed to identify a threshold for the lower number of reads for the minor DNA components to call for the presence of the corresponding species and to set up a more precise quantitative analysis. This is also important to distinguish background contamination from the real presence of minor quantity of one species. Contamination from one run to another could be a potential problem for NGS technologies and appropriate monitoring approaches should be used [20]. In addition, DNA extraction, PCR and emulsion PCR should be carried out in different rooms to avoid accidental contaminations between samples.

To optimize the efficiency to map reads (and indirectly also improve the sensitivity of the assays), we tested different alignment algorithms and evaluated the error rate of the semiconductor sequencing technology on the amplified mtDNA regions. Of the three reported algorithms, SW and *mem* performances, evaluated based on the number of read mapped, were comparable on these Ion Torrent data. These two algorithms are known to tolerate more errors in the alignments and over these two, *mem* has been reported as the most appropriate algorithm for Ion Torrent sequencing reads [43]. In our hands, *mem* worked better for the 16S_KH fragment (the shortest fragment among the three considered regions) as the number of non filtered and filtered reads was the same (S4 Table), confirming its efficiency on Ion Torrent data. Other algorithms and approaches already described for other purposes and applications [44, 45] could be tested to evaluate if other mappers and bioinformatics strategies could be more efficient in terms of percentage of mapped reads. Computing time was not a big problem as in a laptop with 4 Gbytes of RAM, mapping work with *mem* lasted about 5 minutes. Compared to the metagenomic approach described by Ripp et al. [24] that sequenced all extracted DNA without any pre-amplification step of target DNA regions, our approach does not need any computing facilities and data can be easily handled and analysed in a few minutes, making our approach suitable for small labs that do not have access to powerful computing facilities. Error rates (S4 and S5 Tables) were comparable to what was reported in a NGS study that used the Roche 454 Junior sequencing technology for species identification with the 16S gene (on average: 0.0014 per base [20]). Error rates did not introduce any bias in both studies (our study and [20]) that could prevent the correct assignment of reads to the corresponding species.

## Conclusions

In this work, we showed that the Ion Torrent NGS technology can be applied for identification of mammalian and avian species in DNA mixtures. The strategy that we tested was to include more than one primer pair to amplify informative mtDNA regions. This approach can overcome potential problems of amplification competition among species or PCR failure derived by other reasons (i.e. partial inhibition of PCR derived by poor DNA extraction from complex food products). Several other primer pairs can be tested to evaluate their efficiency as universal primers, including primers specifically designed for avian species. This benchtop technology, that can produce a large number of reads in a short time (sequencing step can last about 3–4 hours), have the potential to be applied in routine assays for species determination not only in meat samples or meat-based products but also in many other applied fields in which this information is needed.

## Supporting Information

S1 Fig Alignments of the mtDNA target regions amplified with primers 12S_KH reported in Table 2. The grey parts are the primer regions.
(JPG)

S2 Fig Alignments of the mtDNA target regions amplified with primers 16S_KH reported in Table 2. The grey parts are the primer regions.
(JPG)

S3 Fig Alignments of the mtDNA target regions amplified with primers 16S_Ki reported in Table 2. The grey parts are the primer regions.
(JPG)

S4 Fig Phylogenetic relationships of the mtDNA region amplified with primer pair 12S_KH reported in Table 2. (JPG)

S5 Fig Phylogenetic relationships of the mtDNA region amplified with primer pair 16S_KH reported in Table 2. (JPG)

S6 Fig Phylogenetic relationships of the mtDNA region amplified with primer pair 16S_Ki reported in Table 2. (JPG)

S1 Table mtDNA sequences used as reference. (DOCX)

S2 Table Amplified regions of the mtDNA according to the reference sequences reported in S1 Table. (DOCX)

S3 Table Differences between sequences obtained from databases and those obtained by Sanger sequencing. (DOCX)

S4 Table Number of aligned reads and error rates per species and primer pair combinations obtained from library 1. (DOCX)

S5 Table Error rates calculated per species across the three amplified regions. (DOCX)

S6 Table Number of reads and their percentage among species obtained from libraries 2, 3A and 3B. (DOCX)
