# Supplementary figures and images for: A Next Generation Semiconductor Based Sequencing Approach for the Identification of Meat Species in DNA Mixtures
Source: PLoS One. 2015 Apr 29;10(4):e0121701. doi: 10.1371/journal.pone.0121701 (PMC4414512; doi:10.1371/journal.pone.0121701)

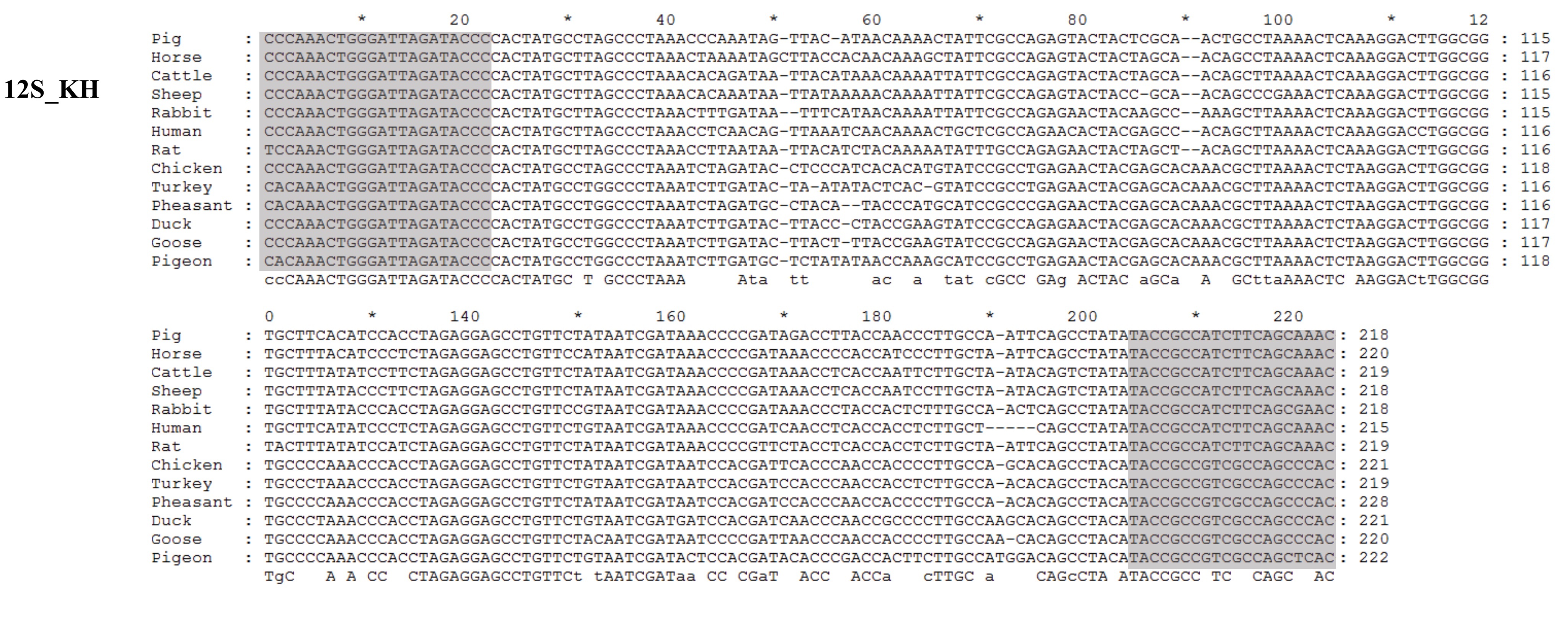

Supplement: S1 Fig — The grey parts are the primer regions. (JPG) [file pone.0121701.s001.jpg]

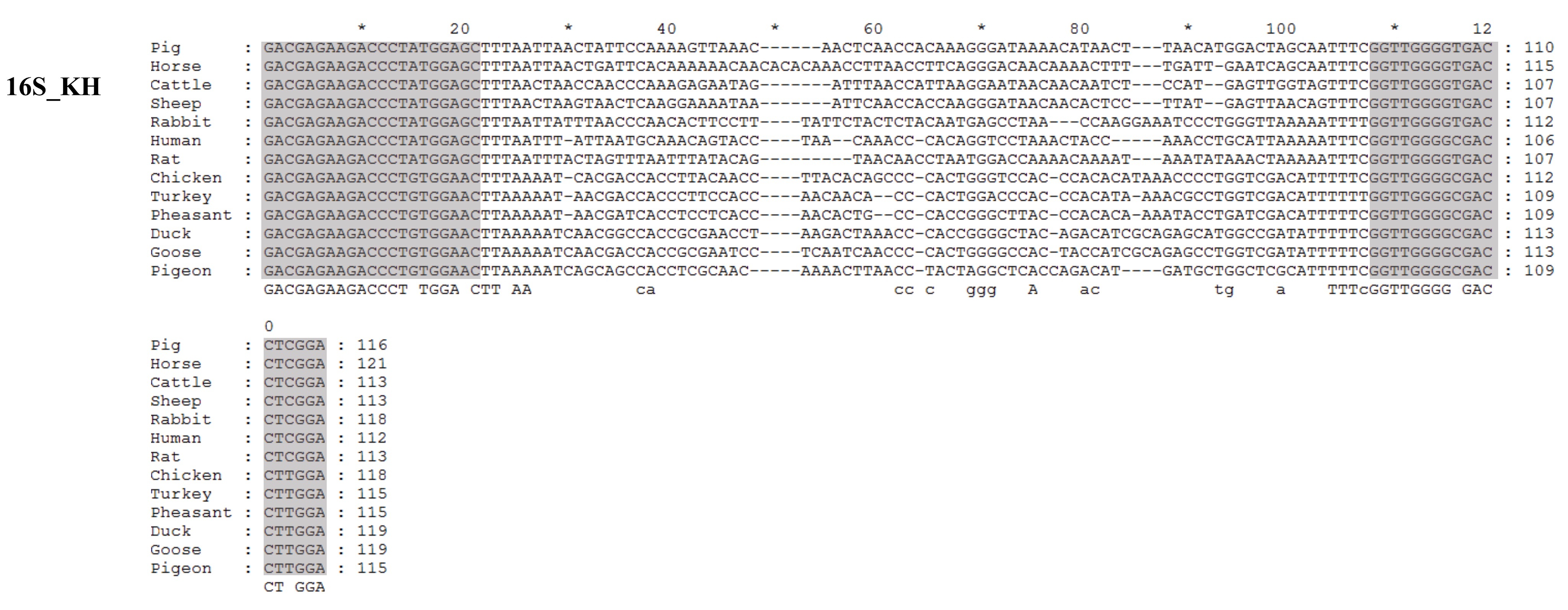

Supplement: S2 Fig — The grey parts are the primer regions. (JPG) [file pone.0121701.s002.jpg]

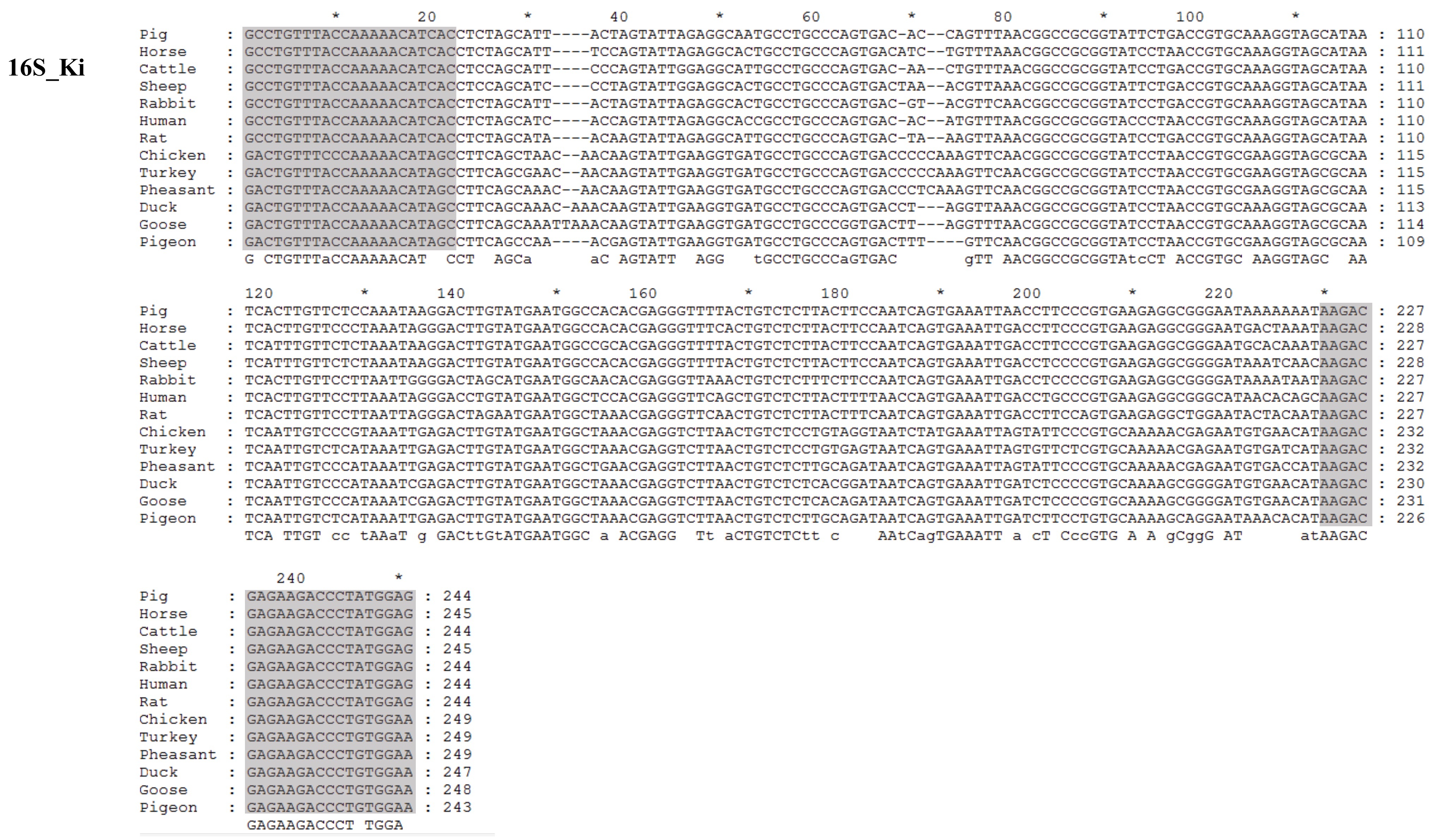

Supplement: S3 Fig — The grey parts are the primer regions. (JPG) [file pone.0121701.s003.jpg]

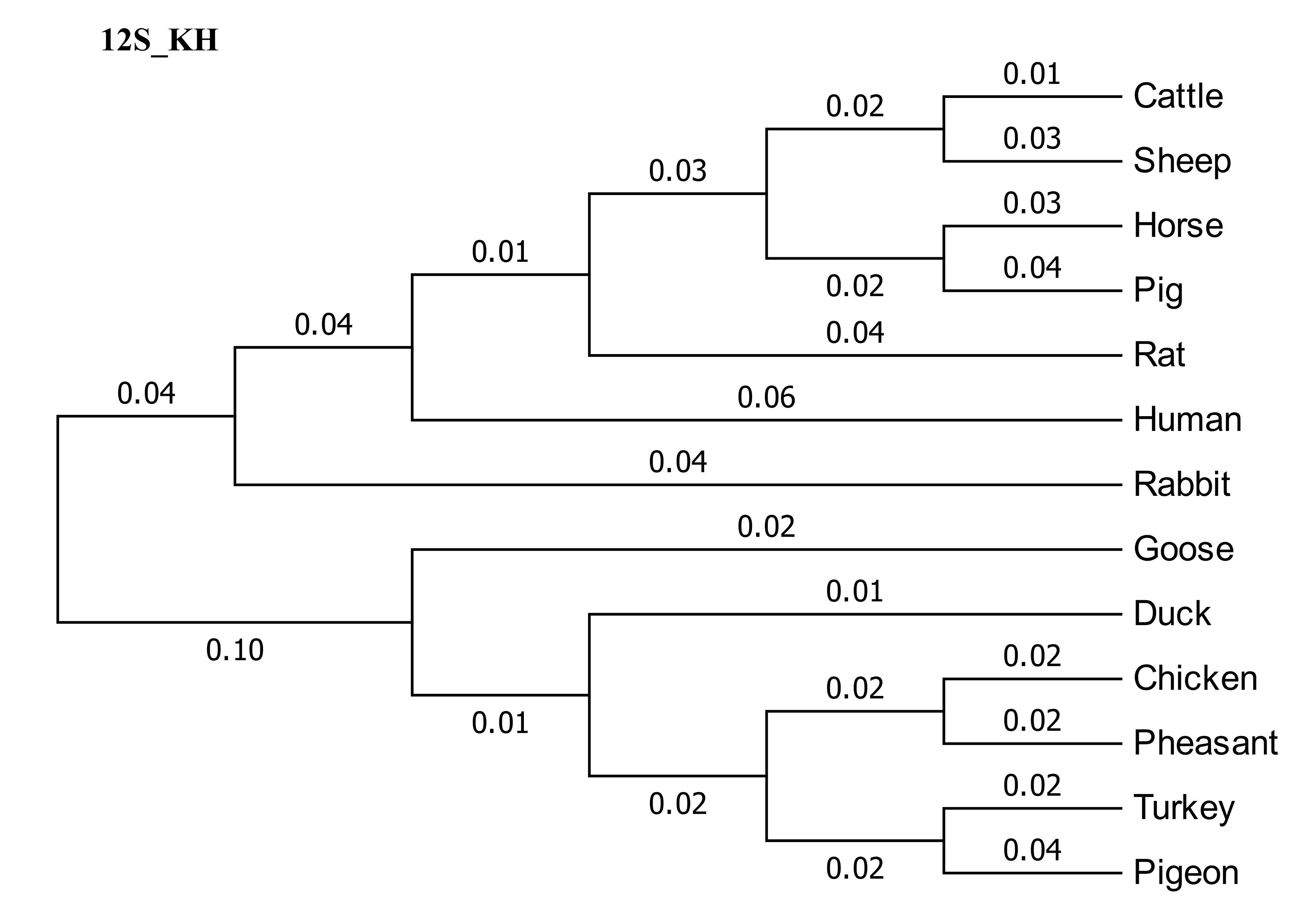

Supplement: S4 Fig — (JPG) [file pone.0121701.s004.jpg]

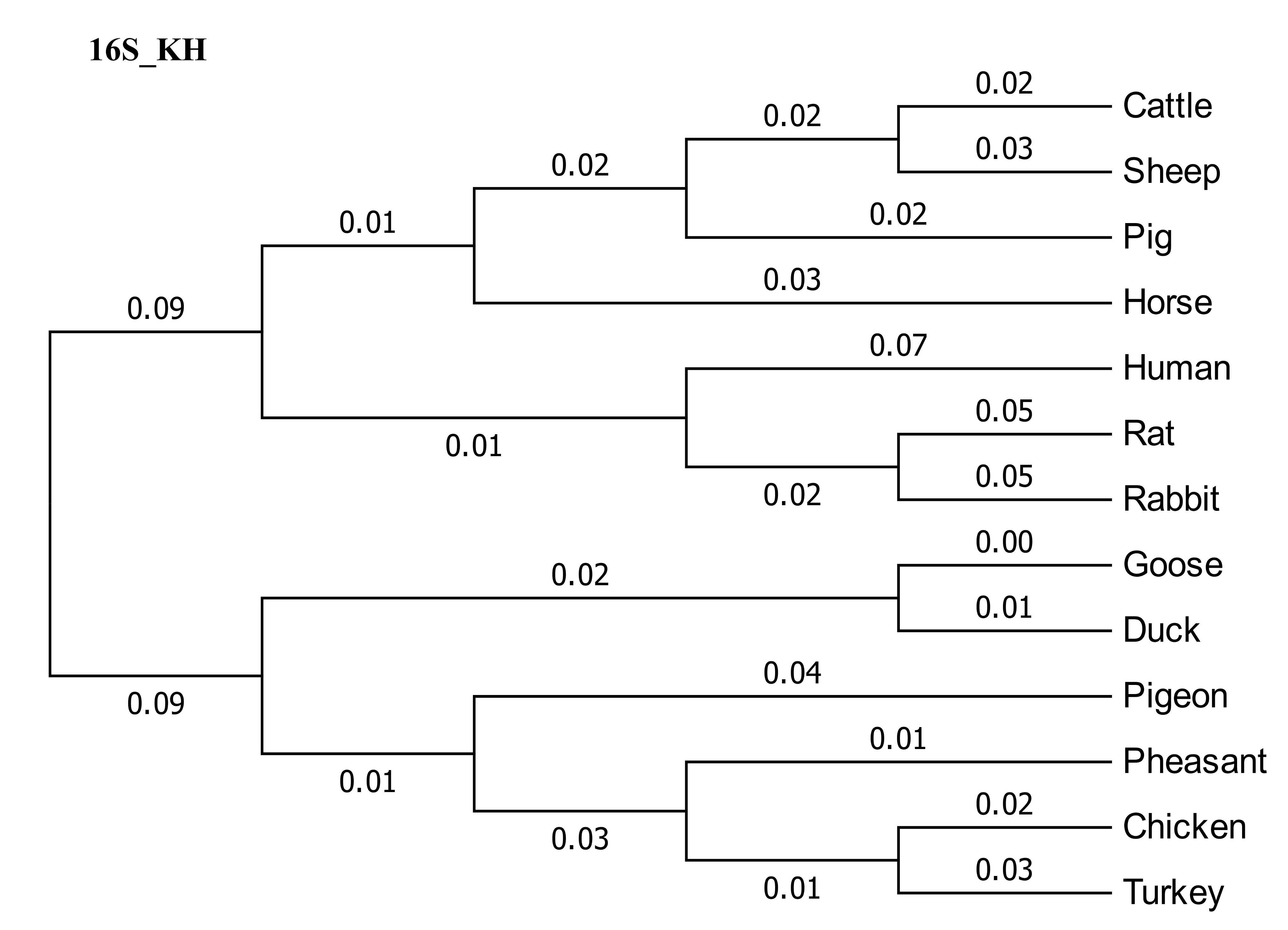

Supplement: S5 Fig — (JPG) [file pone.0121701.s005.jpg]

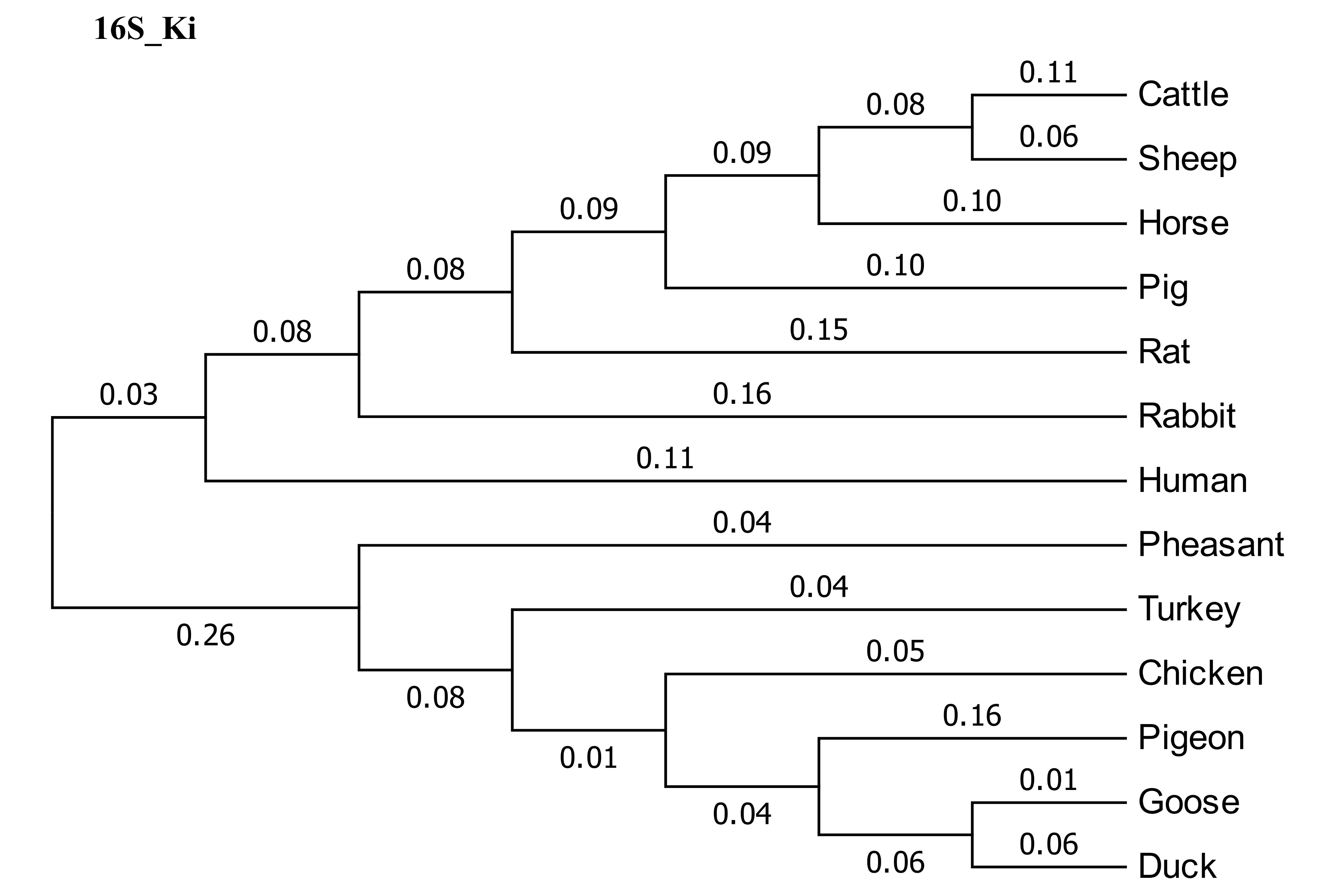

Supplement: S6 Fig — (JPG) [file pone.0121701.s006.jpg]
