# Supplementary material for: A Next Generation Semiconductor Based Sequencing Approach for the Identification of Meat Species in DNA Mixtures
Source: PLoS One. 2015 Apr 29;10(4):e0121701. doi: 10.1371/journal.pone.0121701 (PMC4414512; doi:10.1371/journal.pone.0121701)
Supplement: S1 Table — (DOCX) [file pone.0121701.s007.docx]

**S1 Table.** **mtDNA sequences used as reference.**

| **Species** | **Database** | **Sequence version** | **Accession number** |
| --- | --- | --- | --- |
| Pig (*Sus scrofa domesticus*) | Ensembl | Sscrofa10.2 | GCA_00003025.4 |
| Horse (*Equus caballus*) | Ensembl | EquCab2 | GCA_000002305.1 |
| Cattle (*Bos taurus*) | Ensembl | UMD3.1 | GCA_000003055.3 |
| Sheep (*Ovies aries*) | Ensembl | Oarv3.1 | GCA_000298735.1 |
| Rabbit (*Oryctolagus cuniculus*) | Ensembl | OryCun2.0 | GCA_000003625.1 |
| Human (*Homo sapiens*) | Ensembl | GRCh38 | GCA_000001405.15 |
| Rat (*Rattus rattus*) | GenBank | - | NC_012374.1 |
| Chicken (*Gallus gallus domesticus*) | Ensembl | Galgal4 | GCA_000002315.2 |
| Turkey *(Meleagris gallopavo*) | Ensembl | UMD2 | GCA_000146605.1 |
| Pheasant (*Phasianus colchicus*) | GenBank | - | FJ752430.1 |
| Duck (*Anas platyrhynchos domesticus*) | GenBank | - | KJ883269.1 |
| Goose (*Anser anser*) | GenBank | - | EU932689.1 |
| Pigeon (*Columba livia*) | GenBank | - | KJ722068.1 |
