# Supplementary material for: A Next Generation Semiconductor Based Sequencing Approach for the Identification of Meat Species in DNA Mixtures
Source: PLoS One. 2015 Apr 29;10(4):e0121701. doi: 10.1371/journal.pone.0121701 (PMC4414512; doi:10.1371/journal.pone.0121701)
Supplement: S2 Table — (DOCX) [file pone.0121701.s008.docx]

**S2 Table.** **Amplified regions of the mtDNA according to the reference sequences reported in S1 Table.**

| **Amplified regions** | **Species** | **Amplified regions of the reference mtDNA (including primers)** | **Amplified regions of the reference mtDNA (excluding primers)** | **Amplified fragments (including primers; in bp)** | **Amplified fragments (excluding primers; in bp)** |
| --- | --- | --- | --- | --- | --- |
| 12S_KH | Pig | 1660-1877 | 1682-1857 | 218 | 176 |
|  | Horse | 489-708 | 511-688 | 220 | 178 |
|  | Cattle | 840-1058 | 862-1038 | 219 | 177 |
|  | Sheep | 482-699 | 504-679 | 218 | 176 |
|  | Rabbit | 486-703 | 508-683 | 218 | 176 |
|  | Human | 1064-1278 | 1086-1258 | 215 | 173 |
|  | Rat | 484-702 | 506-661 | 219 | 177 |
|  | Chicken | 1726-1946 | 1748-1926 | 221 | 179 |
|  | Turkey | 498-716 | 520-696 | 219 | 177 |
|  | Pheasant | 1636-1854 | 1658-1834 | 219 | 177 |
|  | Duck | 1566-1786 | 1588-1766 | 221 | 179 |
|  | Goose | 521-740 | 543-720 | 220 | 178 |
|  | Pigeon | 497-718 | 519-698 | 222 | 180 |
| 16S_KH | Pig | 3322-3437 | 3343-3419 | 116 | 77 |
|  | Horse | 2168-2288 | 2189-2270 | 121 | 82 |
|  | Cattle | 2511-2623 | 2532-2605 | 113 | 74 |
|  | Sheep | 2154-2266 | 2175-2248 | 113 | 74 |
|  | Rabbit | 2147-2264 | 2168-2246 | 118 | 79 |
|  | Human | 2716-2827 | 2737-2809 | 112 | 73 |
|  | Rat | 1921-2257 | 2166-2239 | 113 | 74 |
|  | Chicken | 3425-3542 | 3446-3524 | 118 | 79 |
|  | Turkey | 2192-2306 | 2213-2288 | 115 | 76 |
|  | Pheasant | 3332-3446 | 3353-3428 | 115 | 76 |
|  | Duck | 3239-3357 | 3260-3339 | 119 | 80 |
|  | Goose | 2195-2313 | 2216-2295 | 119 | 80 |
|  | Pigeon | 2168-2282 | 2189-2264 | 115 | 76 |
| 16S_Ki | Pig | 3098-3341 | 3120-3319 | 244 | 200 |
|  | Horse | 1943-2187 | 1965-2165 | 245 | 201 |
|  | Cattle | 2287-2530 | 2309-2508 | 244 | 200 |
|  | Sheep | 1929-2173 | 1951-2151 | 245 | 201 |
|  | Rabbit | 1923-2166 | 1945-2144 | 244 | 200 |
|  | Human | 2492-2735 | 2514-2713 | 244 | 200 |
|  | Rat | 1929-2172 | 1941-2128 | 244 | 200 |
|  | Chicken | 3196-3444 | 3218-3422 | 249 | 205 |
|  | Turkey | 1963-2211 | 1985-2189 | 249 | 205 |
|  | Pheasant | 3103-3351 | 3125-3329 | 249 | 205 |
|  | Duck | 3012-3258 | 3034-3236 | 247 | 203 |
|  | Goose | 1967-2214 | 1989-2192 | 248 | 204 |
|  | Pigeon | 1945-2187 | 1967-2165 | 243 | 199 |
