# Supplementary material for: A Next Generation Semiconductor Based Sequencing Approach for the Identification of Meat Species in DNA Mixtures
Source: PLoS One. 2015 Apr 29;10(4):e0121701. doi: 10.1371/journal.pone.0121701 (PMC4414512; doi:10.1371/journal.pone.0121701)
Supplement: S3 Table — (DOCX) [file pone.0121701.s009.docx]

**S3 Table.** **Differences between sequences obtained from databases and those obtained by Sanger sequencing.**

| **Amplified regions** | **Species** | **Number of variants identified with Sanger sequencing** | **Variants** |
| --- | --- | --- | --- |
| 12S_KH | Pig | 0 |  |
|  | Horse | 0 |  |
|  | Cattle | 0 |  |
|  | Sheep | 1 | m.565_566insG |
|  | Rabbit | 1 | m.656C>T |
|  | Human | 0 |  |
|  | Rat | 0 |  |
|  | Chicken | 0 |  |
|  | Turkey | 0 |  |
|  | Pheasant | 0 |  |
|  | Duck | 0 |  |
|  | Goose | 0 |  |
|  | Pigeon | 0 |  |
| 16S_KH | Pig | 0 |  |
|  | Horse | 0 |  |
|  | Cattle | 0 |  |
|  | Sheep | 0 |  |
|  | Rabbit | 0 |  |
|  | Human | 0 |  |
|  | Rat | 0 |  |
|  | Chicken | 0 |  |
|  | Turkey | 0 |  |
|  | Pheasant | 0 |  |
|  | Duck | 0 |  |
|  | Goose | 0 |  |
|  | Pigeon | 0 |  |
| 16S_Ki | Pig | 0 |  |
|  | Horse | 1 | m.2226delT |
|  | Cattle | 0 |  |
|  | Sheep | 0 |  |
|  | Rabbit | 0 |  |
|  | Human | 0 |  |
|  | Rat | 0 |  |
|  | Chicken | 0 |  |
|  | Turkey | 0 |  |
|  | Pheasant | 0 |  |
|  | Duck | 0 |  |
|  | Goose | 0 |  |
|  | Pigeon | 0 |  |
