# Supplementary material for: A Next Generation Semiconductor Based Sequencing Approach for the Identification of Meat Species in DNA Mixtures
Source: PLoS One. 2015 Apr 29;10(4):e0121701. doi: 10.1371/journal.pone.0121701 (PMC4414512; doi:10.1371/journal.pone.0121701)
Supplement: S4 Table — (DOCX) [file pone.0121701.s010.docx]

**S4 Table.** **Number of aligned reads and error rates per species and primer pair combinations obtained from library 1.**

| **Amplified fragment** | **Species** | **Raw alignment (no. of reads)** | | | **Filtered alignments (no. of reads)** | | | **Mean lenght of aligned reads^1^** | **Total no. of aligned bases** | **Total no. of errors** | **Error rate** |
| --- | --- | --- | --- | --- | --- | --- | --- | --- | --- | --- | --- |
|  |  | ***aln*** | ***SW*** | ***mem*** | ***aln*** | ***SW*** | ***mem*** |  |  |  |  |
| 12S_KH | Pig | 80 | 428 | 446 | 80 | 378 | 443 | 139 | 61680 | 159 | 0.0026 |
|  | Horse | 99 | 186 | 185 | 99 | 172 | 182 | 133 | 24346 | 198 | 0.0081 |
|  | Cattle | 202 | 346 | 445 | 201 | 292 | 377 | 151 | 56789 | 293 | 0.0052 |
|  | Sheep | 324 | 897 | 918 | 323 | 693 | 869 | 157 | 136794 | 207 | 0.0015 |
|  | Rabbit | 417 | 664 | 681 | 417 | 640 | 680 | 164 | 111911 | 237 | 0.0021 |
|  | Human | 527 | 935 | 866 | 527 | 823 | 851 | 157 | 133805 | 187 | 0.0014 |
|  | Rat | 927 | 1017 | 1023 | 825 | 963 | 1002 | 168 | 168758 | 300 | 0.0012 |
|  | Chicken | 92 | 222 | 187 | 87 | 137 | 180 | 119 | 21496 | 166 | 0.0078 |
|  | Turkey | 67 | 552 | 577 | 67 | 493 | 561 | 147 | 82731 | 62 | 0.0007 |
|  | Pheasant | 297 | 809 | 819 | 291 | 692 | 798 | 119 | 95585 | 146 | 0.0015 |
|  | Duck | 284 | 985 | 981 | 277 | 926 | 928 | 157 | 146333 | 163 | 0.0011 |
|  | Goose | 430 | 1039 | 1024 | 425 | 894 | 751 | 147 | 110541 | 188 | 0.0017 |
|  | Pigeon | 944 | 1420 | 1425 | 941 | 1353 | 1419 | 157 | 223566 | 214 | 0.0010 |
| 16S_KH | Pig | 266 | 504 | 526 | 266 | 304 | 526 | 76 | 40017 | 93 | 0.0023 |
|  | Horse | 22 | 145 | 180 | 22 | 65 | 180 | 78 | 14107 | 146 | 0.0103 |
|  | Cattle | 273 | 464 | 486 | 273 | 428 | 486 | 71 | 34638 | 28 | 0.0008 |
|  | Sheep | 187 | 335 | 341 | 187 | 248 | 341 | 72 | 24513 | 66 | 0.0027 |
|  | Rabbit | 161 | 304 | 308 | 161 | 271 | 308 | 76 | 23493 | 38 | 0.0017 |
|  | Human | 72 | 165 | 162 | 72 | 140 | 162 | 60 | 9679 | 30 | 0.0031 |
|  | Rat | 383 | 381 | 387 | 314 | 351 | 387 | 78 | 30191 | 56 | 0.0019 |
|  | Chicken | 600 | 763 | 776 | 600 | 710 | 776 | 76 | 60142 | 47 | 0.0008 |
|  | Turkey | 75 | 192 | 206 | 75 | 155 | 206 | 74 | 15214 | 122 | 0.0080 |
|  | Pheasant | 785 | 959 | 974 | 785 | 899 | 974 | 75 | 73374 | 22 | 0.0003 |
|  | Duck | 630 | 819 | 829 | 630 | 738 | 829 | 78 | 64984 | 46 | 0.0007 |
|  | Goose | 654 | 840 | 861 | 654 | 671 | 861 | 78 | 67505 | 79 | 0.0012 |
|  | Pigeon | 233 | 485 | 494 | 232 | 355 | 494 | 71 | 34956 | 128 | 0.0037 |
| 16S_Ki | Pig | 299 | 584 | 595 | 297 | 555 | 578 | 170 | 98684 | 278 | 0.0028 |
|  | Horse | 201 | 601 | 633 | 201 | 535 | 553 | 182 | 100792 | 191 | 0.0028 |
|  | Cattle | 211 | 622 | 606 | 210 | 568 | 584 | 174 | 102051 | 204 | 0.0020 |
|  | Sheep | 92 | 330 | 343 | 92 | 287 | 308 | 175 | 53927 | 228 | 0.0042 |
|  | Rabbit | 224 | 493 | 496 | 224 | 465 | 481 | 172 | 83194 | 160 | 0.0019 |
|  | Human | 240 | 676 | 672 | 240 | 650 | 664 | 181 | 120370 | 226 | 0.0182 |
|  | Rat | 372 | 426 | 427 | 312 | 407 | 424 | 181 | 76785 | 139 | 0.0018 |
|  | Chicken | 18 | 39 | 41 | 18 | 31 | 29 | 185 | 5371 | 52 | 0.0097 |
|  | Turkey | 3 | 11 | 9 | 3 | 4 | 2 | 205 | 410 | 1 | 0.0024 |
|  | Pheasant | 47 | 129 | 132 | 47 | 119 | 125 | 179 | 22381 | 176 | 0.0079 |
|  | Duck | 74 | 156 | 150 | 74 | 92 | 72 | 164 | 11840 | 50 | 0.0042 |
|  | Goose | 89 | 216 | 223 | 87 | 115 | 108 | 176 | 19015 | 183 | 0.0096 |
|  | Pigeon | 1 | 2 | 8 | 1 | 1 | 1 | 199 | 199 | 2 | 0.0101 |

^1^ Rounded numbers.
