# Supplementary material for: A Next Generation Semiconductor Based Sequencing Approach for the Identification of Meat Species in DNA Mixtures
Source: PLoS One. 2015 Apr 29;10(4):e0121701. doi: 10.1371/journal.pone.0121701 (PMC4414512; doi:10.1371/journal.pone.0121701)
Supplement: S5 Table — (DOCX) [file pone.0121701.s011.docx]

**S5 Table. Error rates calculated per species across the three amplified regions.**

| **Species** | **Error rate** |
| --- | --- |
| Pig | 0.003 |
| Horse | 0.004 |
| Cattle | 0.003 |
| Sheep | 0.002 |
| Rabbit | 0.002 |
| Human | 0.002 |
| Rat | 0.002 |
| Chicken | 0.003 |
| Turkey | 0.002 |
| Pheasant | 0.002 |
| Duck | 0.001 |
| Goose | 0.002 |
| Pigeon | 0.001 |
