# Supplementary material for: A Next Generation Semiconductor Based Sequencing Approach for the Identification of Meat Species in DNA Mixtures
Source: PLoS One. 2015 Apr 29;10(4):e0121701. doi: 10.1371/journal.pone.0121701 (PMC4414512; doi:10.1371/journal.pone.0121701)
Supplement: S6 Table — (DOCX) [file pone.0121701.s012.docx]

**S6 Table. Number of reads and their percentage among species obtained from libraries 2, 3A, and 3B.**

| **Target mtDNA regions** | **Species/Mean - SD - Total no. Reads** | **Library 2 number of reads (Mammalian + Avian amplifications)** | **Library 2 in percentage on the whole library (Mammalian + Avian)** | **Library 2 (percentage of reads separated for the two classes)** | **Library 2 (number of reads normalized: 1 ng of DNA)** | **Library 3A** | **Library 3B** | **Libraries (3A + 3B): Total no. of reads of libraries 3A and 3B** | **Libraries (3A + 3B)/2: mean number of reads for the two libraries 3** | **Libraries [(3A + 3B)/2]: no. of read normalized – 1 ng of DNA** | **Libraries (3A + 3B)/2 in percentage on the whole library (Mammalian + Avian)** | **Libraries (3A + 3B)/2 in percentage distinguished for the two classes** |
| --- | --- | --- | --- | --- | --- | --- | --- | --- | --- | --- | --- | --- |
| 12S_KH | Pig | 695 | 0.07 | 0.11 | 121.93 | 268 | 826 | 1094 | 547 | 182.33 | 0.11 | 0.13 |
|  | Horse | 1025 | 0.11 | 0.17 | 179.82 | 55 | 62 | 117 | 58.5 | 19.50 | 0.01 | 0.01 |
|  | Cattle | 1396 | 0.15 | 0.23 | 244.91 | 1169 | 929 | 2098 | 1049 | 349.67 | 0.20 | 0.25 |
|  | Sheep | 149 | 0.02 | 0.02 | 26.14 | 138 | 108 | 246 | 123 | 41.00 | 0.02 | 0.03 |
|  | Rabbit | 1464 | 0.15 | 0.24 | 256.84 | 1278 | 1017 | 2295 | 1147.5 | 382.50 | 0.22 | 0.27 |
|  | Human | 1394 | 0.15 | 0.23 | 244.56 | 1229 | 1255 | 2484 | 1242 | 414.00 | 0.24 | 0.29 |
|  | Rat | 71 | 0.01 | 0.01 | 12.46 | 60 | 73 | 133 | 66.5 | 22.17 | 0.01 | 0.02 |
|  | **Mean** | **884.86** |  |  | **155.24** | **599.57** | **610.00** | **1209.57** | **604.79** | **201.60** |  |  |
|  | **sd** | **593.78** |  |  | **104.17** | **590.39** | **511.66** | **1071.31** | **535.65** | **178.55** |  |  |
|  | **Total n. of reads (Mammalian)** | **6194** | **0.65** | **1.00** | **1086.67** | **4197** | **4270** | **8467** | **4233.5** | **1411.17** | **0.82** | **1.00** |
|  | Chicken | 90 | 0.01 | 0.03 | 13.43 | 29 | 242 | 271 | 135.5 | 45.17 | 0.03 | 0.15 |
|  | Turkey | 693 | 0.07 | 0.21 | 103.43 | 167 | 280 | 447 | 223.5 | 74.50 | 0.04 | 0.24 |
|  | Pheasant | 536 | 0.06 | 0.16 | 80.00 | 100 | 81 | 181 | 90.5 | 30.17 | 0.02 | 0.10 |
|  | Duck | 335 | 0.04 | 0.10 | 50.00 | 78 | 44 | 122 | 61 | 20.33 | 0.01 | 0.07 |
|  | Goose | 1112 | 0.12 | 0.33 | 165.97 | 276 | 186 | 462 | 231 | 77.00 | 0.04 | 0.25 |
|  | Pigeon | 592 | 0.06 | 0.18 | 88.36 | 126 | 222 | 348 | 174 | 58.00 | 0.03 | 0.19 |
|  | **Mean** | **559.67** |  |  | **83.53** | **129.33** | **175.83** | **305.17** | **152.58** | **50.86** |  |  |
|  | **sd** | **345.11** |  |  | **51.51** | **85.44** | **93.63** | **139.06** | **69.53** | **23.18** |  |  |
|  | **Total n. of reads (Avian)** | **3358** | **0.35** | **1.00** | **501.19** | **776** | **1055** | **1831** | **915.5** | **305.17** | **0.18** | **1.00** |
|  | **Total n. of reads (M+A)** | **9552** | **1.00** |  | **1587.86** | **4973** | **5325** | **10298** | **5149** | **1716.33** | **1.00** |  |
| 16S_KH | Pig | 387 | 0.09 | 0.18 | 67.89 | 415 | 488 | 903 | 451.5 | 150.50 | 0.13 | 0.15 |
|  | Horse | 26 | 0.01 | 0.01 | 4.56 | 34 | 37 | 71 | 35.5 | 11.83 | 0.01 | 0.01 |
|  | Cattle | 869 | 0.19 | 0.41 | 152.46 | 1538 | 1112 | 2650 | 1325 | 441.67 | 0.39 | 0.44 |
|  | Sheep | 111 | 0.02 | 0.05 | 19.47 | 169 | 123 | 292 | 146 | 48.67 | 0.04 | 0.05 |
|  | Rabbit | 415 | 0.09 | 0.20 | 72.81 | 724 | 530 | 1254 | 627 | 209.00 | 0.19 | 0.21 |
|  | Human | 276 | 0.06 | 0.13 | 48.42 | 445 | 290 | 735 | 367.5 | 122.50 | 0.11 | 0.12 |
|  | Rat | 41 | 0.01 | 0.02 | 7.19 | 39 | 66 | 105 | 52.5 | 17.50 | 0.02 | 0.02 |
|  | **Mean** | **303.57** |  |  | **53.26** | **480.57** | **378.00** | **858.57** | **429.29** | **143.10** |  |  |
|  | **sd** | **295.22** |  |  | **51.79** | **528.28** | **378.67** | **902.95** | **451.48** | **150.49** |  |  |
|  | **Total n. of reads (Mammalian)** | **2125** | **0.47** | **1.00** | **372.81** | **3364** | **2646** | **6010** | **3005** | **1001.67** | **0.89** | **1.00** |
|  | Chicken | 424 | 0.09 | 0.18 | 63.28 | 82 | 166 | 248 | 124 | 41.33 | 0.04 | 0.34 |
|  | Turkey | 181 | 0.04 | 0.08 | 27.01 | 46 | 34 | 80 | 40 | 13.33 | 0.01 | 0.11 |
|  | Pheasant | 475 | 0.10 | 0.20 | 70.90 | 44 | 39 | 83 | 41.5 | 13.83 | 0.01 | 0.12 |
|  | Duck | 263 | 0.06 | 0.11 | 39.25 | 37 | 18 | 55 | 27.5 | 9.17 | 0.01 | 0.08 |
|  | Goose | 696 | 0.15 | 0.29 | 103.88 | 109 | 84 | 193 | 96.5 | 32.17 | 0.03 | 0.27 |
|  | Pigeon | 364 | 0.08 | 0.15 | 54.33 | 33 | 28 | 61 | 30.5 | 10.17 | 0.01 | 0.08 |
|  | **Mean** | **400.50** |  |  | **59.78** | **58.50** | **61.50** | **120.00** | **60.00** | **20.00** |  |  |
|  | **sd** | **179.91** |  |  | **26.85** | **30.27** | **56.04** | **80.48** | **40.24** | **13.41** |  |  |
|  | **Total n. of reads (Avian)** | **2403** | **0.53** | **1.00** | **358.66** | **351** | **369** | **720** | **360** | **120.00** | **0.11** | **1.00** |
|  | **Total n. of reads (M+A)** | **4528** | **1.00** |  | **731.46** | **3715** | **3015** | **6730** | **3365** | **1121.67** | **1.00** |  |
| 16S_Ki | Pig | 704 | 0.07 | 0.08 | 123.51 | 692 | 1261 | 1953 | 976.5 | 325.50 | 0.07 | 0.07 |
|  | Horse | 135 | 0.01 | 0.01 | 23.68 | 135 | 262 | 397 | 198.5 | 66.17 | 0.01 | 0.01 |
|  | Cattle | 2340 | 0.23 | 0.25 | 410.53 | 4948 | 2577 | 7525 | 3762.5 | 1254.17 | 0.25 | 0.25 |
|  | Sheep | 246 | 0.02 | 0.03 | 43.16 | 550 | 327 | 877 | 438.5 | 146.17 | 0.03 | 0.03 |
|  | Rabbit | 1965 | 0.19 | 0.21 | 344.74 | 3864 | 2342 | 6206 | 3103 | 1034.33 | 0.21 | 0.21 |
|  | Human | 3577 | 0.35 | 0.39 | 627.54 | 7141 | 4500 | 11641 | 5820.5 | 1940.17 | 0.39 | 0.39 |
|  | Rat | 279 | 0.03 | 0.03 | 48.95 | 241 | 710 | 951 | 475.5 | 158.50 | 0.03 | 0.03 |
|  | **Mean** | **1320.86** |  |  | **231.73** | **2510.14** | **1711.29** | **4221.43** | **2110.71** | **703.57** |  |  |
|  | **sd** | **1327.36** |  |  | **232.87** | **2803.54** | **1535.48** | **4311.90** | **2155.95** | **718.65** |  |  |
|  | **Total n. of reads (Mammalian)** | **9246** | **0.91** | **1.00** | **1622.11** | **17571** | **11979** | **29550** | **14775** | **4925** | **1.00** | **1.00** |
|  | Chicken | 10 | 0.00 | 0.01 | 1.49 | 0 | 0 | 0 | 0 | 0.00 | 0.00 | 0.00 |
|  | Turkey | 258 | 0.03 | 0.30 | 38.51 | 1 | 1 | 2 | 1 | 0.33 | 0.00 | 1.00 |
|  | Pheasant | 235 | 0.02 | 0.27 | 35.07 | 0 | 0 | 0 | 0 | 0.00 | 0.00 | 0.00 |
|  | Duck | 59 | 0.01 | 0.07 | 8.81 | 0 | 0 | 0 | 0 | 0.00 | 0.00 | 0.00 |
|  | Goose | 226 | 0.02 | 0.26 | 33.73 | 0 | 0 | 0 | 0 | 0.00 | 0.00 | 0.00 |
|  | Pigeon | 79 | 0.01 | 0.09 | 11.79 | 0 | 0 | 0 | 0 | 0.00 | 0.00 | 0.00 |
|  | **Mean** | **144.50** |  |  | **21.57** | **0.17** | **0.17** | **0.33** | **0.17** | **0.06** |  |  |
|  | **sd** | **107.15** |  |  | **15.99** | **0.41** | **0.41** | **0.82** | **0.41** | **0.14** |  |  |
|  | **Total n. of reads (Avian)** | **867** | **0.09** | **1.00** | **129.40** | **1** | **1** | **2** | **1** | **0.33** | **0.00** | **1.00** |
|  | **Total n. of reads (M+A)** | **10113** | **1.00** |  | **1751.51** | **17572** | **11980** | **29552** | **14776** | **4925.33** | **1.00** |  |
